# Detection of novel *PPP1R1B::STARD3* fusion transcript in acute myeloid leukemia: a case report

**DOI:** 10.1186/s13256-024-04536-w

**Published:** 2024-06-05

**Authors:** Elahe Dehghani Firouzabadi, Mohammed Allami, Eman Jassim Mohammed, Hossein Barzegar, Mahtab Dastpak, Reza Alemohammad, Vahid Moghimi, Reihaneh Alsadat Mahmoudian, Fatemeh Nasrabadi, Nahid Arghiani, Yohei Kitamura, Seyed Abolfazl Hosseini, Ali Ghasemi, Moein Farshchian

**Affiliations:** 1 Stem Cell and Regenerative Medicine Research Group, Academic Center for Education, Culture, and Research (ACECR), Mashhad, Razavi Khorasan Iran; 2 Department of Biology, Faculty of Science, Hakim Sabzevar University, Sabzevar, Iran; 3 Department of Dentistry, Al-Manara College for Medical Sciences, Maysan, Iraq; 4 Department of Biology, Faculty of Science, Ferdowsi University of Mashhad, Mashhad, Iran; 5 Department of Biology, College of Science, Mustansiriyah University, Baghdad, Iraq; 6 Department of Cell Biology, Harvard Medical School, 240 Longwood Ave, Boston, MA 02115 USA; 7 Basic Sciences Research Institute, Mashhad University of Medical Sciences, Mashhad, Iran; 8 Cancer Research Center, Mashhad University of Medical Sciences, Mashhad, Iran; 9 Department of Molecular Biosciences, The Wenner-Gren Institute, Stockholm University, Stockholm, Sweden; 10 Department of Biochemistry and Biomedicine, School of Life Sciences, University of Sussex, Brighton, UK; 11 Department of Neurosurgery, Keio University School of Medicine, Tokyo, Japan; 12 Department of Pediatrics Hematology and Oncology, School of Medicine, Mashhad University of Medical Sciences, Mashhad, Iran

**Keywords:** Fusion transcripts, Acute myeloid leukemia, RNA-seq

## Abstract

**Background:**

Acute myeloid leukemia (AML) is the second most common type of leukemia in children. Although prognostic and diagnostic tests of AML patients have improved, there is still a great demand for new reliable clinical biomarkers for AML. Read-through fusion transcripts (RTFTs) are complex transcripts of adjacent genes whose molecular mechanisms are poorly understood. This is the first report of the presence of the *PPP1R1B::STARD3* fusion transcript in an AML patient. Here, we investigated the presence of *PPP1R1B::STARD3* RTFT in a case of AML using paired-end RNA sequencing (RNA-seq).

**Case presentation:**

A Persian 12-year-old male was admitted to Dr. Sheikh Hospital of Mashhad, Iran, in September 2019 with the following symptoms, including fever, convulsions, hemorrhage, and bone pain. The patient was diagnosed with AML (non-M3-FAB subtype) based on cell morphologies and immunophenotypical features. Chromosomal analysis using the G-banding technique revealed t (9;22) (q34;q13).

**Conclusions:**

Single-cell RNA sequencing (scRNA-seq) analysis suggested that the *PPP1R1B* promoter may be responsible for the *PPP1R1B::STARD3* expression. Alterations in the level of lipid metabolites implicate cancer development, and this fusion can play a crucial role in the cholesterol movement in cancer cells. *PPP1R1B::STARD3* may be considered a candidate for targeted therapies of the cholesterol metabolic and the PI3K/AKT signaling pathways involved in cancer development and progression.

**Supplementary Information:**

The online version contains supplementary material available at 10.1186/s13256-024-04536-w.

## Introduction

Acute myeloid leukemia (AML) is a hematologic malignancy of myeloid progenitor cells that accounts for 15–20% of pediatric leukemias [1]. It is a complex genetic disorder with multiple fusion genes that regulate cell proliferation, differentiation, and apoptosis [2]. Fusion genes are genomic and transcriptional abnormalities associated with these fusion loci that can potentially lead to tumor formation and are utilized as diagnostic, prognostic, and therapeutic targets for AML patients [3, 4]. Currently, there are several techniques for the identification of fusion genes, including fluorescence in situ hybridization (FISH), whole-genome sequencing (WGS), and transcriptome analysis [5, 6]. Transcriptome analysis using the RNA-seq method is the best approach to identify novel fusion or chimeric (the transcripts generated via gene fusion and trans-splicing) transcripts for AML diagnosis [7–9]. The *PPP1R1B::STARD3* is a chimeric transcript generated via RNA processing resulting from the splicing of two adjacent pre-mRNAs in the same direction without chromosomal abnormalities [10–12]. It has been indicated that the frequency of *PPP1R1B::STARD3* is approximately 21.3% and 8.3% in gastric and breast cancer patients, respectively [11, 12]. This chimeric RNA is the product of a read-through event between the *PPP1R1B* and *STARD3* genes separated by 455 bp at chr17:q12 [12]. The fusion site is usually between exon 6 of *PPP1R1B* and exon 2 of *STARD3* [11–13]. Protein Phosphatase 1 Regulatory (Inhibitor) Subunit 1B (*PPP1R1B*), as a dopamine and cAMP regulated phosphoprotein 32 kDa (DARPP-32), was discovered in the brain and plays a key role in brain signaling and physiological processes. Moreover, t-DARPP, the truncated splice isoform, is expressed in tumor cells of gastric, breast, prostate, colon, and stomach cancers [12, 14–17]. The StAR-related lipid transfer protein domain 3 (*STARD3*), as a member of the steroidogenic acute regulatory-related lipid transfer (START) protein family, plays a critical role in the transfer of lipids through both vesicular and non-vesicular pathways. Due to the importance of lipid metabolism in cancer cells, any change in the expression of these metabolites may contribute to cancer progression [18]. Overexpression of *PPP1R1B::STARD3* may increase cancer cell proliferation and tumorigenesis by activating the PI3K/AKT pathway [12].

Here, we identified a *PPP1R1B::STARD3* fusion transcript in a 12-year-old Iranian boy with AML by transcriptome analysis and confirmed it using q-PCR and Sanger sequencing, suggesting that it may be a novel biomarker in AML. Moreover, single-cell RNA sequencing (scRNA-seq) analysis was used to infer the expression pattern of *STARD3* and *PPP1R1B* subclones according to different AML cell populations and normal samples using public scRNA-seq expression matrix data.

## Case presentation

A Persian 12-year-old male was admitted to Dr. Sheikh Hospital of Mashhad, Iran, in September 2019 with the following symptoms, including fever, convulsions, hemorrhage, and bone pain. The peripheral blood examination determined 173.4 K/L white blood cells (WBC) with 86% blast cells, 10 g/dl hemoglobin (Hb), and 19 × 10^9/L platelets. The bone marrow aspiration revealed hypercellularity and a notable decrease in the number of mature myeloid cells and megakaryocytes in the presence of 75% blast cells. Immunophenotyping of the bone marrow sample was carried out by a flow cytometer (Attune NxT, ThermoFisher, USA), and data were analyzed using FlowJo v7.6 software (Tree Star, Ashland, OR). The cells were positive for CD45 (95%), HLA-DR (76%), CD33 (41%), and CD34 (94%) markers, while they were negative for CD19 (< 1%), CD3 (8%), CD5 (< 1%), CD10 (3%), CD20 (< 1%), CD61 (< 1%), CD71 (3%), and CD117 (< 1%) markers. The patient was diagnosed with AML (non-M3-FAB subtype) based on cell morphologies and immunophenotypical features. Bone marrow evaluation revealed an abnormal male chromosome complement composite karyotype with translocation between chromosomes 9 and 22 in 15 metaphases of 15 cells examined (t (9;22)(q34; q13)). The patient received induction chemotherapy. However, he died a few months after the diagnosis.

### RNA sequencing, RT-PCR, and Sanger sequencing

**Fig. 1:**
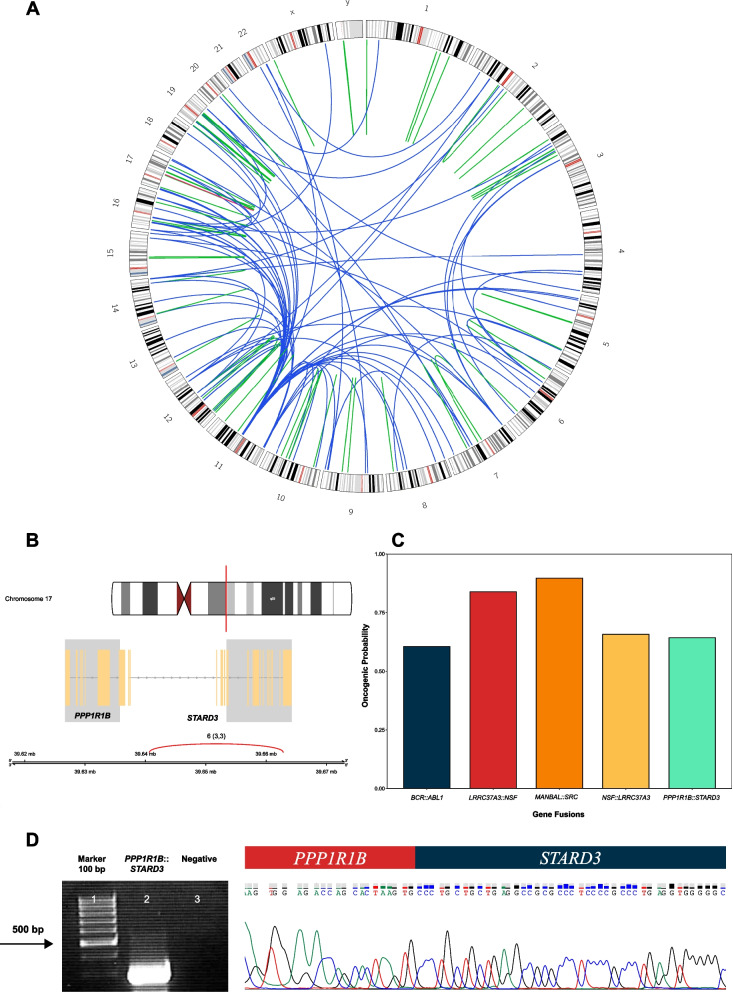
**A** Circular genomic landscape of detected fusion transcripts in acute myeloid leukemia case using FusionCatcher. The intrachromosomal fusion transcripts have been indicated with blue links, while the interchromosomal ones are connected as green ribbons. *Protein phosphatase 1 regulatory (inhibitor) subunit 1B*::*StAR-related lipid transfer protein domain 3 *has been marked with a red colored ribbon. **B** Fusion plot of *Protein phosphatase 1 regulatory (inhibitor) subunit 1B*::*StAR-related lipid transfer protein domain 3* indicating the position of both partner genes involved in the fusion event on chromosome 17. The red link indicates the breakpoint between two partner genes with the number of sequencing reads, which supports the fusion event. **C** Bar plot of the oncogenic probability of different candidate fusions predicted by DEEPrior. The oncogenicity of *Protein phosphatase 1 regulatory (inhibitor) subunit 1B*::*StAR-related lipid transfer protein domain 3* was predicted by 64%. **D** Detection of *Protein phosphatase 1 regulatory (inhibitor) subunit 1B*::*StAR-related lipid transfer protein domain 3* fusion transcript using RT-PCR. Lane 1: size marker (100 bp ladder); lane 2: patient sample; lane 3: negative control. RT-PCR analysis for the *Protein phosphatase 1 regulatory (inhibitor) subunit 1B*::*StAR-related lipid transfer protein domain 3* fusion transcript showed a 293 bp band on the agarose gel. Sanger sequencing confirmed fusion between the Protein phosphatase 1 regulatory (inhibitor) subunit 1B and StAR-related lipid transfer protein domain 3 genes

**Fig. 2:**
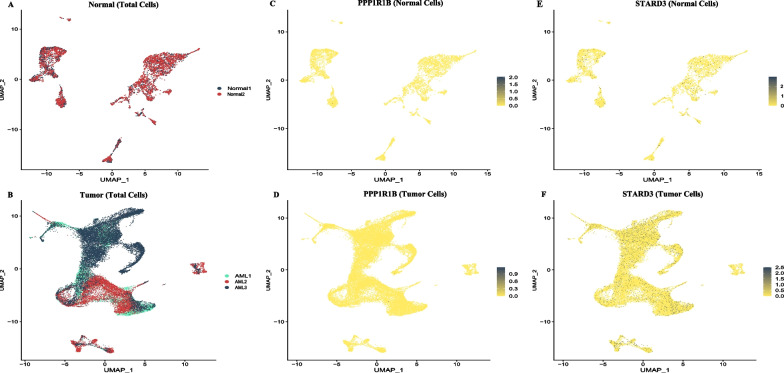
Visualization of Single-cell RNA sequencing data. The Single-cell RNA sequencing data consists of 35,000 acute myeloid leukemia cells and 6200 normal cells on three acute myeloid leukemia and two normal samples. **A **and** B** Uniform manifold approximation and projection show the merged single-cell transcriptomes for normal and tumor cells. **C **and** D** Expression of Protein phosphatase 1 regulatory (inhibitor) subunit 1B for normal and tumor cells, respectively. **E **and** F** Expression of StAR-related lipid transfer protein for normal and tumor cells, respectively

Total RNA was extracted from the bone marrow mononuclear cells (BMMNCs) using the Tripure reagent (Roche, Mannheim, Germany) according to the manufacturer's instructions. The RNA-seq libraries were prepared using a KAPA HyperPrep kit with RiboErase (HMR) and RNA-seq was performed with 100 million paired reads on the NovaSeq 6000 platform (Illumina) (CeGaT company, Tubingen, Germany). FusionCatcher software v1.20 [19], a Python-based tool, was exploited to investigate the fusion transcripts, identifying read-through fusion transcripts (RTFTs) between *PPP1R1B* (NM_032192.4) and *STARD3* (NM_006804.4) transcripts. A complete list of fusions is provided in Additional File 1: Table S1. An overview of all 234 fusion events detected by Circos, a software package for data visualization [20], as well as the location of both genes involved in the *PPP1R1B::STARD3* fusion on chromosome 17 using the chimeraviz package v1.24.0 [21] in R is shown in Fig. 1A, B. To further confirm this finding, we analyzed an RNA-seq data profile (GSE142514) for 35 samples of AML and identified the *PPP1R1B::STARD3* fusion (2.8%). A complete list of fusion transcripts identified by FusionCatcher is available in Additional File 2: Table S2. The oncogenicity of fusions was assessed by DEEPrior v2.0 [22], a deep learning technique, to investigate the amino acid sequences of the fused proteins (Additional File 3: Table S3). The oncogenic potential of the *PPP1R1B::STARD3* was predicted in 64% of fusions, and the oncogenic probability of the top selected candidate fusions was depicted via a bar plot using ggplot2 package v3.4.0 [23] in R (Fig. 1C). The detection of *PPP1R1B::STARD3* fusion transcript (*PPP1R1B* forward primer 5′-TCTGGATGAGTCCGAGAGAGA-3′ and *STARD3* reverse primer 5′- GTCGAAGGTGACGAAGAGACA-3′) was validated by RT-PCR and Sanger sequencing (Fig. 1D). To gain further insight into the exclusivity of the *PPP1R1B::STARD3* fusion*,* we analyzed two RNA-seq data profiles (including GSE115525 and PRJNA589314) for B-cell acute lymphoblastic leukemia (B-ALL) samples. Interestingly, there are no reports of the presence of *PPP1R1B::STARD3* in B-ALL patients was found in previous studies and RNA-seq data. Moreover, the expression levels of *PPP1R1B* and *STARD3* were investigated through scRNA-seq matrices for three AML and two normal bone marrow samples (phs000159) obtained from dbGap [24]. After quality control, 35,000 AML cells and 6200 normal cells were identified for further analysis. Principal component analysis (PCA) was applied for the variable genes, and the uniform manifold approximation and projection (UMAP) algorithm was applied for dimensionality reduction and visualization (Seurat implementation) (Fig. 2).

## Results and discussion

 In cancer, it is not uncommon for two or more neighbor genes to be co-transcribed into a single mRNA, which may leads to production of a read-through fusion transcripts (RTFT) and potentially, a corresponding fusion protein. These fusion transcripts may contain some or all exons from two adjacent genes, where the transcription start site is from the upstream gene and the termination site is from the downstream gene [25, 26]. RTFTs leads to dysregulation of oncogenes by upregulating of downstream genes, resulting in the development of various types of cancer [27–29]. Abnormalities of chromosome 17 in AML patients are associated with poor prognosis and chemotherapy resistance [30–35]. This study introduced *PPP1R1B::STARD3* as a novel fusion transcript in an Iranian AML patient using RNA-seq analysis, which identified the *PPP1R1B::STARD3* fusion transcript and found that this fusion breakpoint is located at a different site compared to previous reports. *STARD3* plays a pivotal role in cholesterol transfer in cancer cells, leading to cancer cell proliferation and development. Its ectopic expression can increase tumor aggressiveness in ovarian and breast cancers, suggesting its potential role in cancer treatment [36, 37]. On the other hand, *PPP1R1B* is involved in gastric and pancreatic tumorigenesis [17, 30, 38–42]. *PPP1R1B* binds BCL-2 and calcineurin, which contributes to the anti-apoptotic function of BCL-2 through reducing the inositol 1,4,5-triphosphate receptor (InsP3R) phosphorylation [14, 43]. Evidence suggests that aberrant expression of *PPP1R1B::STARD3* significantly increases cell proliferation through the PI3K/AKT signaling pathway [12, 18]. Dysregulation of the PI3K/AKT pathway is involved in numerous human cancers, including colorectal, breast, and hematologic malignancies, indicating the therapeutic value of this pathway in cancer treatment [44, 45]. The UMAP analysis demonstrated that a higher proportion of AML samples expressed *STARD3* compared to normal samples. Nevertheless, there was no significant detection of *PPP1R1B* expression in either normal or AML samples. The results suggest that the *PPP1R1B::STARD3* fusion transcript may be expressed due to abnormal activation of the *PPP1R1B* promoter. The expression of *PPP1R1B::STARD3* in solid tumors (gastric and breast cancers) and leukaemia, but not B-ALL, shows that this fusion transcript may play an essential role in cancer development through the PI3K-AKT pathway. Taken together, our data may suggest that *PPP1R1B::STARD3* contributes to tumorigenesis and may be a prognostic marker in AML patients with the potential to develop therapeutic targets.

## Conclusion

In summary, our data elucidate the first report of the *PPP1R1B::STARD3* fusion transcript, confirmed by Sanger sequencing, in an Iranian AML patient, which may be a valuable target for AML diagnosing and treatment. This fusion may avail as a new potential and therapeutic biomarker for AML. However, further analysis is required to investigate the oncogenic function of the *PPP1R1B::STARD3* fusion genes and the downstream molecular events due to this fusion transcript.

### Supplementary Information

**Additional file 1: **A complete list of fusions identified by FusionCatcher (following ID SRR18012668).

**Additional file 2: **A complete list of fusions identified by FusionCatcher (following ID GSE142514).

**Additional file 3: **Detailed report of DEEPrior for AML patient (following ID SRR18012668).

## Data Availability

Raw RNA sequencing data is publicly available in NCBI under the following ID SRR18012668, and the *PPP1R1B::STARD3* fusion transcript breakpoint was deposited in the GenBank at the NCBI under accession number OL695927.

## Abbreviations

AML: Acute myeloid leukemia
RTFTs: Read-through fusion transcripts
FISH: Fluorescence in situ hybridization
WGS: Whole-genome sequencing
PPP1R1B: Protein phosphatase 1 regulatory (inhibitor) subunit 1B
DARPP-32: Dopamine and cAMP regulated phosphoprotein 32 kDa
STARD3: StAR-related lipid transfer protein domain 3
START: Steroidogenic acute regulatory-related lipid transfer
scRNA-seq: Single-cell RNA sequencing
WBC: White blood cell
Hb: Hemoglobin
BMMNCs: Bone marrow mononuclear cells
PCA: Principal component analysis
UMAP: Uniform manifold approximation and projection
B-ALL: B-cell acute lymphoblastic leukemia
InsP3R: Inositol 1,4,5-triphosphate receptor
